# Effect of extracellular vesicles on malignant tumours: Mechanisms and clinical findings (Review)

**DOI:** 10.3892/ol.2026.15857

**Published:** 2026-09-11

**Authors:** Ruiyi Shen, Qihong Lan, Yuan Lu, Ke Wen, Zhenzhen Wei, Qian Liu

**Affiliations:** Graduate School, Integrated Chinese and Western Medicine Institute for Children Health and Drug Innovation, Jiangxi University of Chinese Medicine, Nanchang, Jiangxi 330004, P.R. China

**Keywords:** extracellular vesicles, malignant tumours, intercellular communication, tumour microenvironment

## Abstract

Extracellular vesicles (EVs) are critical mediators of intercellular communication within the tumour microenvironment (TME). EVs carry bioactive cargo, including proteins, nucleic acids and lipids, that modulate tumourigenesis, progression and therapeutic resistance via a range of molecular and cellular mechanisms. As natural carriers of functional biomolecules, EVs contribute to key oncogenic processes, including tumour cell proliferation, invasion, immune evasion and drug resistance. Notably, EVs secreted by cancer cells harbour tumour-specific molecular signatures, such as oncoproteins, non-coding RNAs and mutant transcripts, thereby facilitating microenvironmental reprogramming and malignant evolution. The present review systematically examines five key molecular mechanisms by which tumour-derived EVs promote malignant progression: i) Remodelling of the extracellular matrix and organ-specific metastasis through the delivery of metastasis-associated proteins such as matrix metalloproteinases and integrins; ii) induction of immune cell exhaustion and tumour immune evasion through the release of immunosuppressive molecules, including programmed death ligand 1 and transforming growth factor-β; iii) establishment of tumour neovascular networks through the delivery of pro-angiogenic factors such as vascular endothelial growth factor; iv) reprogramming of the metabolic landscape of the TME through the encapsulation of key enzymes involved in glycolysis and lipid metabolism; and v) mediation of resistance to chemotherapy and targeted therapy through the transport of ATP-binding cassette transporters, inhibitor of apoptosis proteins and other molecules. On this basis, the present review focuses on the unique advantages of EVs as novel liquid biopsy biomarkers for early tumour diagnosis, therapeutic efficacy monitoring and prognostic evaluation, as well as the application prospects of engineered EVs as targeted drug delivery systems for precision tumour therapy. The core conclusion is that the major bottlenecks currently restricting the clinical translation of EVs include the lack of standardised isolation and identification methods, the unclear regulatory mechanisms underlying EV heterogeneity and the low efficiency of targeted *in vivo* delivery. Future research should focus on dissecting the functional specificity of EV subpopulations and performing precision-engineering modifications of EVs, thereby providing new strategic directions for overcoming tumour therapeutic resistance and immune evasion.

## 1. Introduction

Cancer is a highly complex and intrinsically heterogeneous disease. Despite substantial advances in conventional therapeutic modalities, tumour recurrence, metastasis and acquired resistance to anticancer agents remain major clinical obstacles. These persistent challenges have driven intensive investigation into the molecular and cellular mechanisms underlying tumourigenesis and disease progression, with the aim of identifying novel therapeutic targets and strategies. The tumour microenvironment (TME), often conceptualised as the ‘soil’ that supports tumour growth and evolution, has emerged as a critical determinant of cancer biology and treatment response (1). Accumulating evidence indicates that cancer-derived extracellular vesicles (EVs) actively remodel the TME and facilitate key oncogenic processes (2), including immune evasion, angiogenesis and pre-metastatic niche formation (3).

EVs are a heterogeneous population of lipid bilayer-enclosed membrane vesicles that are actively secreted by virtually all cell types and are ubiquitously present in biological fluids, including blood, urine, saliva and cerebrospinal fluid (4). As endogenous biological nanocarriers, EVs selectively package and transport diverse bioactive cargoes, including proteins, nucleic acids [such as mRNA, microRNA (miR/miRNA) and long non-coding RNA] and lipids, enabling intercellular communication through targeted delivery to recipient cells (5). During tumourigenesis and cancer progression, malignant cells secrete increased quantities of EVs enriched with tumour-associated antigens, oncoproteins and oncogenic nucleic acids. These EV-derived molecular signatures closely mirror the pathological and phenotypic features of their parental cells and collectively contribute to key hallmarks of cancer, including sustained proliferative signalling, invasion and metastasis, and evasion of immune surveillance (6,7).

EVs derived from cancer cells serve as pivotal mediators of TME remodelling and tumour progression. Proteins and other bioactive molecules encapsulated within these EVs regulate key oncogenic processes, including tumour cell proliferation, invasion, distant metastasis, immune evasion and therapeutic resistance, through diverse direct and indirect mechanisms (8). For example, tumour-derived EVs can impair immune cell function, thereby facilitating immune evasion. They also contribute to the establishment of pre-metastatic niches by preconditioning distant organ microenvironments to support subsequent colonisation by cancer cells (9,10).

Conventional nanocarrier systems, including liposomes and polymeric nanoparticles, frequently encounter major limitations, such as suboptimal biocompatibility, rapid opsonisation and immune clearance, inadequate tumour-specific targeting and insufficient intratumoural distribution. In recent years, EVs, as endogenous and naturally evolved nanoscale carriers, have emerged as a promising platform for overcoming these challenges (11). Owing to their intrinsic biocompatibility, low immunogenicity, and inherent capacity for tissue penetration and cellular uptake, EVs are increasingly recognised as a highly promising next-generation system for tumour-targeted drug delivery (12). Consequently, EV-based delivery strategies represent a rapidly advancing frontier in oncology therapeutics research.

**Figure 1.**
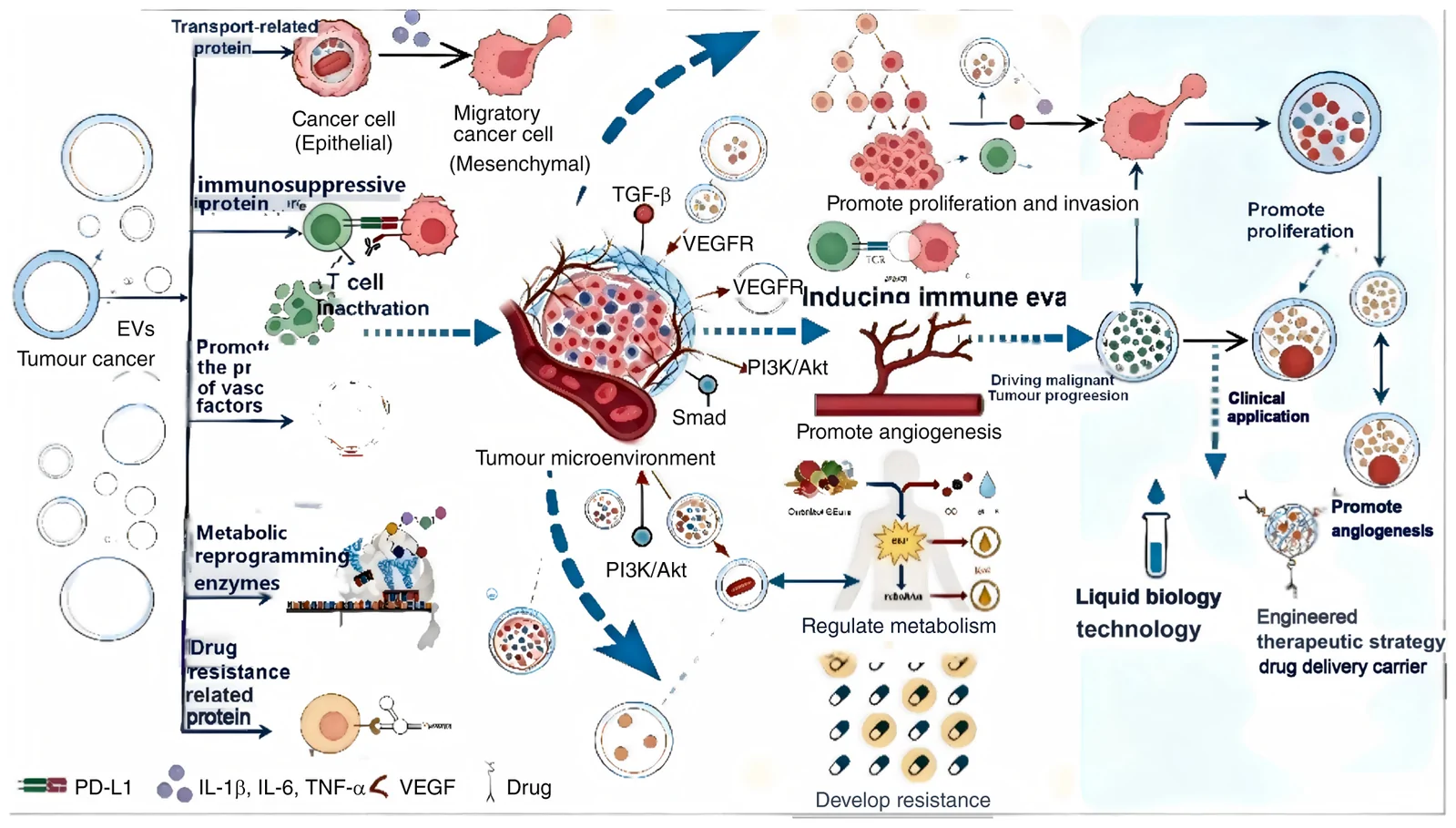
Schematic illustration of the roles of EVs in malignant tumour progression and clinical translation. EVs promote malignant tumour progression by regulating five core biological processes: Tumour proliferation and invasion, immune evasion, angiogenesis, metabolic reprogramming and drug resistance. They also exhibit clinical potential in liquid biopsy-based diagnoses and engineered drug delivery. EV, extracellular vesicle; TGF-βR, transforming growth factor-β receptor; VEGF, vascular endothelial growth factor; VEGFR, VEGF receptor; PD-L1, programmed cell death ligand 1; TNF-α, tumour necrosis factor-α. Created using BioRender.

Therefore, systematically elucidating the specific roles, underlying molecular mechanisms and key functional molecules mediated by EVs in tumour malignancy has pivotal scientific and translational importance. This line of research not only advances the fundamental understanding of cancer biology, shifting the conceptual framework from the classical ‘cancer cell-autonomous’ paradigm to a more integrative ‘tumour ecosystem’ perspective, but also opens promising avenues for clinical translation. Specifically, EVs have substantial potential as non-invasive liquid biopsy biomarkers for early detection and prognostic stratification, as biocompatible targeted drug delivery vehicles with enhanced efficacy and reduced off-target toxicity, and as novel therapeutic targets for intervention. Against this background, the present review makes the following distinctive contributions and presents innovative perspectives. First, by focusing on the protein cargo of EVs, it systematically delineates the molecular mechanisms governing five core malignant processes: Tumour invasion and metastasis, immune evasion, angiogenesis, metabolic reprogramming and therapeutic resistance. The specific functional pathways mediated by distinct functional proteins are also clarified. Second, the review establishes an integrated ‘mechanism-molecule-clinic’ framework that maps previously fragmented research on liquid biopsy biomarkers and engineered EV-based therapeutic strategies to the basic mechanisms elaborated on, thereby elucidating the molecular basis underlying clinical translation. Third, grounded in the functional molecular heterogeneity of EVs, the present review provides an in-depth analysis of the core bottlenecks currently limiting clinical translation and proposes future development directions integrating single-vesicle multi-omics, microfluidic technology and artificial intelligence. The review aims to provide a systematic theoretical reference for basic research in the field of tumour EVs and to identify more targeted research pathways for clinical translation and application (Fig. 1; Table I).

## 2. Core mechanisms underlying the EV-mediated regulation of malignant tumours

### EV-mediated transport of metastasis-associated proteins facilitates tumour invasion

Tumour metastasis is a highly orchestrated, multistep process encompassing local invasion, intravasation, survival in the circulation, extravasation and colonisation of distant organs. A central mechanism underlying this process involves tumour-derived EVs, which facilitate organotropic metastasis and remodel the pre-metastatic niche by selectively delivering bioactive proteins, thereby promoting tumour cell invasion and dissemination (13).

The specific targeting of EVs to distant organs mainly depends on integrin family proteins, such as α6β4 and αvβ5, on the vesicle membrane surface. Integrins are important molecules that mediate cell-extracellular matrix adhesion and confer organ tropism on vesicles. Different integrin heterodimers can be recognised and taken up by vascular endothelial cells in specific target organs. For example, EVs carrying integrin αvβ3 tend to target lung tissue, whereas integrins α6β4 and αvβ5 are associated with liver and brain metastasis, respectively (14). Clinical studies have also confirmed that breast cancer-derived EVs can target and interact with lung tissue endothelial cells by delivering integrin αvβ3, thereby activating local S100 protein expression, promoting formation of a pre-metastatic microenvironment in the lung and creating favourable conditions for the colonisation of circulating tumour cells (15,16).

Following the preliminary formation of the pre-metastatic niche, EVs deliver hydrolases, including matrix metalloproteinases (MMPs). Encapsulated within vesicles and trafficked to the local microenvironment of target organs, these hydrolases specifically degrade extracellular matrix (ECM) structural proteins, disrupt tissue barriers and trigger matrix remodelling, thereby clearing physical obstacles to tumour cell invasion and the establishment of metastatic foci (17). For example, cervical cancer-derived EVs deliver MMP-14 to recipient cells, markedly enhancing ECM remodelling efficiency by activating the ERK signalling pathway and accelerating remodelling of the tumour metastatic microenvironment (18). Furthermore, EV-mediated ECM remodelling itself forms a positive feedback loop that augments the invasive potential of tumour cells. Neuroblastoma-secreted EVs are enriched with a disintegrin and metalloproteinase 10 (ADAM10); after transport to the local microenvironment via vesicles, ADAM10 directly facilitates local invasion and directed migration of tumour cells (19).

Moreover, integrins and the ECM are functionally interdependent rather than independent entities. As pivotal transmembrane receptors, integrins mediate bidirectional communication between cells and the ECM. Integrins facilitate cell-ECM adhesion and transduce extracellular cues into intracellular signalling cascades, thereby critically regulating tumour invasion and metastasis (19). For example, β1 integrin is consistently endogenously upregulated in multiple human malignancies. β1 integrin promotes tumour cell adhesion to specific ECM components (20), such as fibronectin and collagen, and activates downstream effectors, including FAK, Src and PI3K/Akt pathways. Concurrently, it orchestrates cytoskeletal reorganisation through Rho GTPase-mediated actomyosin contractility, collectively enhancing metastatic capacity (21). Furthermore, integrins serve as mechanosensors that detect ECM stiffness, topography and ligand density. Integrins on the surface of EVs also possess mechanosensory functions. This mechanical input is integrated with biochemical signals, particularly through crosstalk with Rho family GTPases, to coordinate polarised cytoskeletal dynamics and directional tumour cell migration (22).

In terms of clinical translation of the aforementioned mechanisms, applications can be categorised into three major dimensions. First, integrin subtypes present on the EV membrane surface can serve as predictive biomarkers for organ-specific metastasis. Supported by the experimental and literature evidence outlined as aforementioned, the endogenous upregulation of integrin αvβ3 indicates an elevated risk of pulmonary metastasis in breast cancer, whereas the natural high expression of integrins α6β4 and αvβ5 is associated with liver and brain metastasis, respectively. In addition, ADAM10 and MMP-14 encapsulated in EVs are closely associated with local tumour invasiveness (23). We propose that this molecular signature can be applied to evaluate preoperative tumour staging and define the extent of surgical resection. Second, targeting metastasis-associated proteins carried by EVs to block formation of the pre-metastatic niche provides a viable direction for therapeutic target selection. Breast cancer-derived EVs drive metastatic progression by delivering integrin αvβ3 (24). We posit that clinical intervention targeting integrin αvβ3 can attenuate metastatic dissemination in breast cancer. Meanwhile, inhibiting MMP-14 secretion and enzymatic activity in EVs can effectively reduce ECM remodelling and local infiltration in cervical cancer. Third, engineered EVs can be rationally modified to achieve lesion-specific drug delivery in clinical settings. Integrin αvβ3-functionalised EVs enable precise delivery of therapeutic agents to pulmonary metastatic lesions, enhancing the pulmonary accumulation efficiency of chemotherapeutic drugs while reducing systemic toxic and adverse effects.

**Figure 2.**
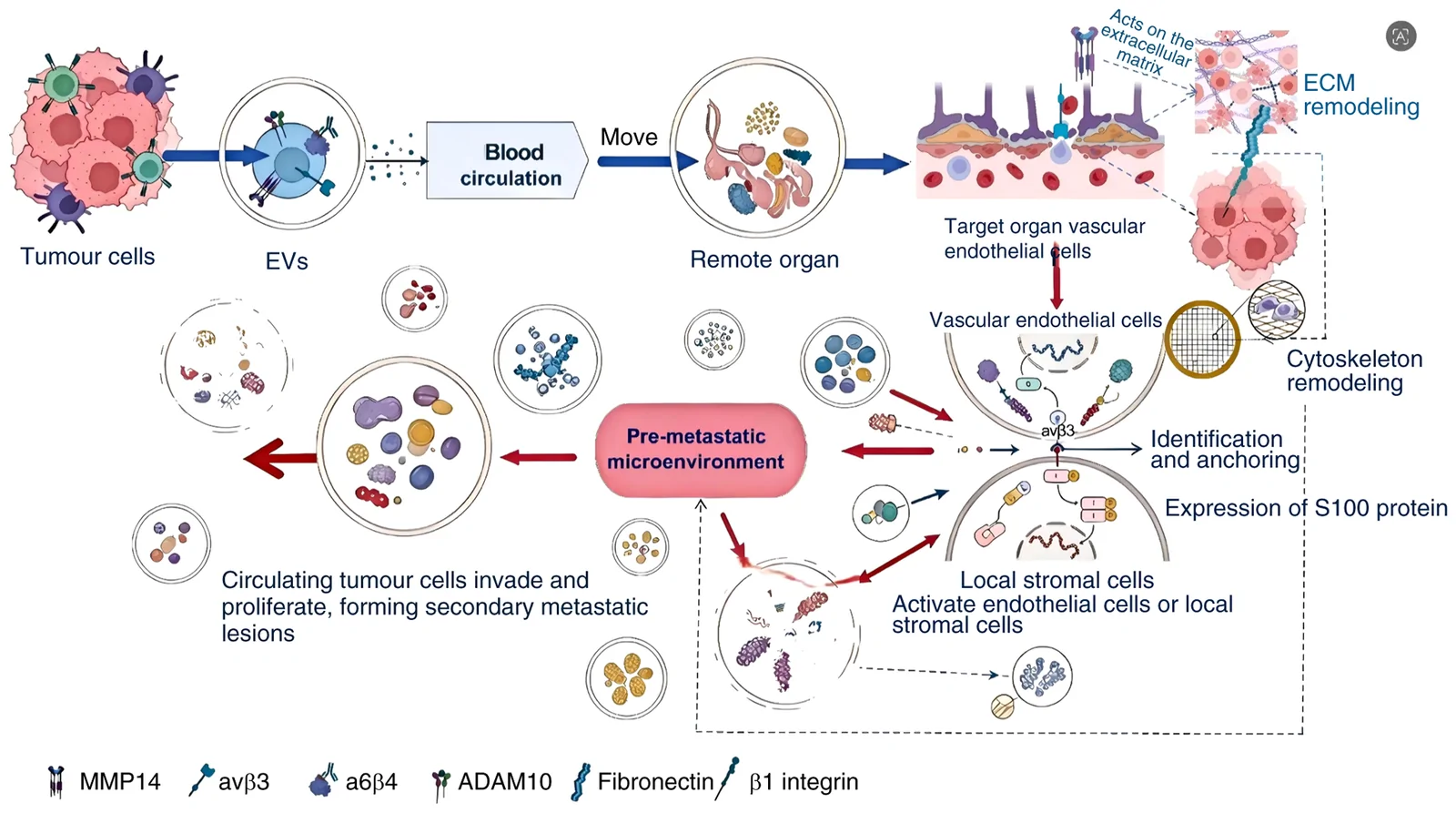
EV-mediated transport of metastasis-associated proteins facilitates tumour invasion. Schematic illustration of the mechanism underlying EV-mediated tumour invasion and pre-metastatic niche formation. Tumour-derived EVs deliver integrins and matrix-degrading enzymes to target organs, induce vascular endothelial activation and ECM remodelling, and establish a pre-metastatic microenvironment conducive to the adhesion and colonisation of CTCs. EV, extracellular vesicle; ECM, extracellular matrix; CTCs, circulating tumour cells; MMP14, matrix metalloproteinase 14; ADAM10, a disintegrin and metalloproteinase domain-containing protein 10. Created using BioRender.

Tumour-derived EVs systemically drive tumour invasion and distant metastasis through a three-tiered mechanistic cascade: Integrin-mediated organ targeting, matrix protease-dependent barrier degradation and integrin-ECM synergistically regulated cell migration. Within this regulatory network, integrin subtypes determine the organ specificity of metastatic dissemination. At present, the differential contributions of distinct EV subpopulations to pre-metastatic niche formation remain poorly defined. Meanwhile, whether integrin-mediated EV targeting is modulated by the physical properties of the TME remains under debate. Detection of specific integrin subtypes in circulating EVs can serve as a predictive biomarker for assessing the risk of tumour metastasis. Small EVs (for example, exosomes) primarily reach distant organs via the systemic circulation, mediate organ-specific targeting via membrane-bound integrins and induce pre-metastatic niche establishment. Disrupting the interaction between EV integrins and endothelial cells of target organs holds promise as a novel strategy to inhibit distant tumour metastasis (Fig. 2).

### EVs carrying immunosuppressive proteins facilitate tumour immune evasion

Tumour immune escape is a multifaceted biological process in which malignant cells evade immune recognition and elimination through a range of molecular and cellular mechanisms, thereby supporting their survival, proliferation and metastatic dissemination. As a hallmark of tumour progression and systemic spread, immune escape involves complex interactions between tumour cells and host immune components. Among the most extensively characterised mechanisms is the secretion of EVs by tumour cells, which carry immunosuppressive cargo, including cytokines, checkpoint ligands and regulatory miRNAs, that actively remodels the TME into an immunosuppressive niche, impairing antitumour immunity and enabling immune evasion (25,26).

One of the best-established mechanisms by which tumour cells evade immune surveillance is the activation of immune checkpoint pathways. Among these, programmed death-ligand 1 (PD-L1) is the most extensively characterised immune checkpoint molecule. PD-L1 is frequently endogenously upregulated at the cell surface in multiple human malignancies, such as non-small-cell lung carcinoma, triple-negative breast cancer, gastric adenocarcinoma and cutaneous melanoma. Upon binding to its cognate receptor programmed cell death protein 1 (PD-1) on activated T lymphocytes, PD-L1 triggers intracellular signalling through the immunoreceptor tyrosine-based inhibitory motif and the immunoreceptor tyrosine-based switch motif (ITSM), leading to recruitment and activation of the inhibitory phosphatase Src homology region 2 domain-containing phosphatase-2. This cascade ultimately suppresses T-cell proliferation, reduces cytokine production and induces functional exhaustion (27). Notably, ITSM phosphorylation is enhanced upon PD-1/PD-L1 engagement, thereby amplifying the inhibitory signal.

EVs secreted by tumour cells serve as critical carriers, enabling PD-L1 to exert systemic immunosuppressive effects. Accumulating evidence indicates that EVs derived from multiple malignant tumours, including melanoma and non-small cell lung cancer, can functionally express PD-L1 on their surface. These PD-L1-bearing EVs bind to PD-1 on the surface of T cells, markedly inhibiting CD8+ T-cell activation and proliferation (25). Meanwhile, PD-L1 signalling induces T-cell apoptosis and blocks T-cell receptor signal transduction. In addition to suppressing T-cell activation, PD-L1 inhibits the PI3K/Akt/mTOR signalling pathway and enhances the expression and function of phosphatase and tensin homologue. It also promotes the differentiation of inducible regulatory T cells (Tregs), which secrete TGF-β to further repress effector T-cell function. These cascading regulatory effects collectively establish systemic immunosuppression, thereby facilitating tumour immune evasion (28).

In the indirect pathway, members of the TGF-β protein family predominantly drive the differentiation of CD4+ T cells into Tregs, which exert potent immunosuppressive effects, mainly through the canonical SMAD signalling cascade (29). This pathway is tightly regulated at multiple hierarchical levels, including ubiquitin-mediated proteasomal degradation of SMAD proteins, phosphatase-catalysed dephosphorylation, and competitive inhibition of receptor-regulated SMADs by inhibitory SMADs (30). Notably, TGF-β has a context-dependent dual role in tumourigenesis: it functions predominantly as a tumour suppressor during early carcinogenesis but frequently acquires pro-tumourigenic properties in advanced disease (31).

EVs modulate immune responses through both direct intercellular communication and indirect signalling pathways, primarily by transferring immunoregulatory molecules such as PD-L1 and TGF-β. Advances in clinical detection methods, such as PD-L1 immunohistochemistry, circulating tumour-DNA sequencing and immune-cell profiling (32), have supported the development of a multilayered immunotherapeutic framework and a comprehensive regulatory network governing immune evasion. Collectively, these findings provide mechanistic insights into tumour-induced immunosuppression and immune escape, as well as a translational basis for the clinical implementation of related diagnostic tools and therapeutic interventions. Specifically, tumour-derived EVs synergistically shape local and systemic immunosuppressive microenvironments through two mechanisms: Direct delivery of PD-L1 to suppress effector T-cell function and indirect delivery of TGF-β to induce Treg differentiation. Furthermore, the intrinsic vesicular carrier properties of EVs markedly prolong the duration and expand the spatial range of action of these immunosuppressive molecules.

**Figure 3.**
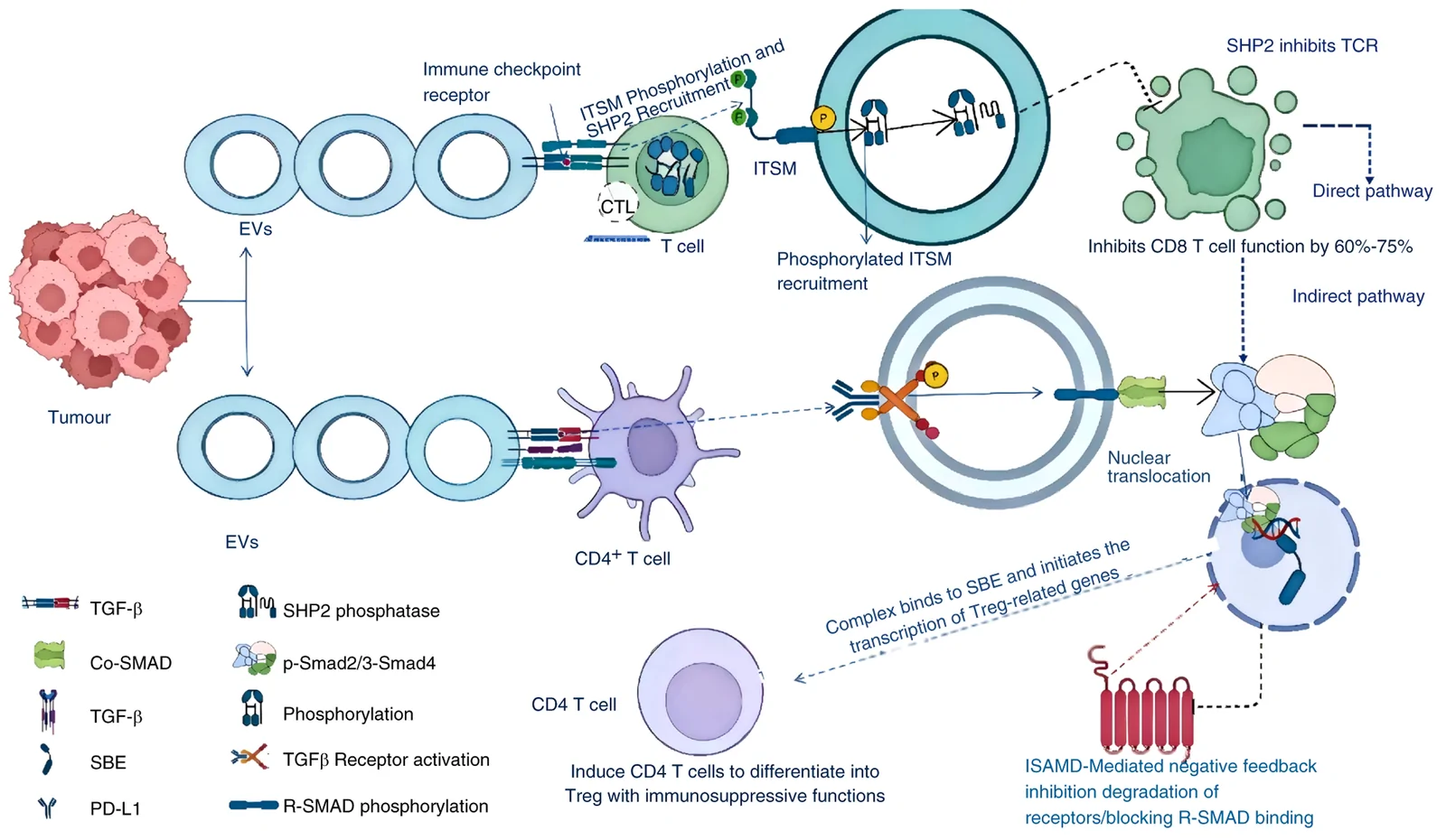
EVs facilitate tumour immune evasion. Schematic illustration of the dual pathways of EV-mediated tumour immune evasion. EVs suppress antitumour immunity through two core pathways. The direct pathway relies on PD-L1 on the EV surface to inhibit the effector functions of CD8+ T cells. The indirect pathway involves EV-derived TGF-β driving the differentiation of CD4+ T cells into immunosuppressive Tregs. EVs, extracellular vesicles; PD-L1, programmed cell death ligand 1; TCR, T-cell receptor; TGF-β, transforming growth factor-β; Tregs, regulatory T cells; SHP2, Src homology 2 domain-containing phosphatase 2; ITSM, immunoreceptor tyrosine-based switch motif; TCR, T-cell receptor; SBE, Smad-binding element; TGFβ receptor, TGF-β receptor; ISAMD, immunoreceptor tyrosine-based switch motif-associated degradation. Created using BioRender.

Nevertheless, the relative contributions of EV-associated PD-L1 and tumour cell surface PD-L1 to immune evasion remain controversial. The active forms of TGF-β and the specific target molecules in EVs from different tumour origins have not been fully elucidated. Meanwhile, circulating EV-associated PD-L1 levels may serve as predictive biomarkers of the therapeutic efficacy of immune checkpoint inhibitors. Bispecific inhibitors simultaneously targeting EV-associated PD-L1 and EV-associated TGF-β may help overcome resistance to single-agent immunotherapy in the future (33) (Fig. 3).

### Angiogenic factors modulate the TME to promote neovascularization and support tumour progression

EV-associated pro-angiogenic factors promote tumour angiogenesis. Tumour growth requires the formation of new blood vessels to supply oxygen and nutrients, a process known as tumour angiogenesis (34). Recent studies have shown that tumour-derived EVs are critical mediators of angiogenesis (35). By delivering multiple pro-angiogenic bioactive factors, EVs help establish a tumour endothelial microenvironment that supports angiogenesis (36).

EVs directly initiate angiogenic programmes by enriching and delivering core factors, including VEGF and basic fibroblast growth factor (bFGF) (37). Among the signalling pathways implicated in this process, the VEGF-VEGFR axis is a central regulatory mechanism governing both angiogenesis and tumour progression (38). Tumour-derived EVs selectively enrich and stabilise VEGF, facilitating its targeted delivery to vascular endothelial cells. This interaction activates pro-angiogenic signalling pathways, thereby promoting endothelial cell proliferation, migration and tubule formation, which are key steps in tumour angiogenesis. Clinical and experimental evidence supports this mechanism. For example, glioblastoma-derived EVs markedly increase VEGF levels in cerebrospinal fluid and enhance the *in vitro* tubulogenesis capacity of human vascular endothelial cells (39).

In addition to VEGF, EVs carry a range of bioactive molecules, including bFGF, platelet-derived growth factor and IL-8 (32). Notably, bFGF binds to its cognate receptor, fibroblast growth factor receptor, to stimulate tumour endothelial cell migration and acts synergistically with VEGF to enhance angiogenesis (40). Furthermore, MMPs packaged within EVs facilitate extracellular matrix degradation, creating permissive microenvironments for neovessel sprouting and stabilisation (36).

EVs enriched in pro-angiogenic factors show an enhanced capacity to stimulate angiogenesis (41). EVs can respond to microenvironmental changes and dynamically modulate the magnitude of angiogenesis. In tumour cells, stress conditions such as hypoxia and oncogene activation upregulate transcription factors, including hypoxia-inducible factor 1α (HIF-1α), leading to increased expression of pro-angiogenic genes such as VEGF and bFGF, thereby altering EV cargo composition (42). Notably, EVs carrying miR-210 suppress key anti-angiogenic regulators, including ephrin-A3, effectively initiating and sustaining the tumour angiogenic switch (43). These molecular mechanisms not only supply tumour cells with essential oxygen and nutrients, but also facilitate local invasion and dissemination into adjacent tissues (44).

This mechanism has substantial translational potential in clinical practice. Clinical evidence indicates that serum EV-associated VEGF levels in patients with colorectal cancer are markedly and positively associated with intratumoural micro vessel density (MVD) scores (45,46). These findings indicate that EV-associated VEGF may serve as a promising non-invasive biomarker for assessing tumour angiogenic activity and predicting patient prognosis, highlighting its potential clinical utility.

From a liquid biopsy perspective, the positive correlation between VEGF levels in serum EVs and tumour tissue MVD suggests that EV-carried VEGF may be useful for evaluating tumour angiogenic status and patient prognosis (47). In therapeutic target screening, the VEGF signalling pathway represents one of the core mechanisms driving tumour neovascularisation (48). Clinical experiments have shown that glioblastoma-derived EVs markedly increase VEGF concentrations in cerebrospinal fluid, exceeding normal levels. Targeting EV-mediated angiogenesis may therefore help overcome resistance induced by conventional anti-VEGF agents. Finally, in the application of engineered EVs, previous studies have shown that EVs loaded with miR-210 can suppress key anti-angiogenic regulators such as ephrin-A3 and effectively trigger the tumour angiogenic switch. Accordingly, miRNAs may be used in engineered EV-based strategies to specifically target tumour endothelial cells (49).

The VEGF signalling pathway constitutes a central regulatory mechanism underlying tumour angiogenesis and is widely recognised as a clinically relevant biomarker in oncology (50,51). Given the pivotal role of VEGF in promoting neovascularisation, therapeutic strategies targeting VEGF or its cognate receptor, VEGFR, have become a major focus in anticancer drug development. Nevertheless, clinical experience indicates that monotherapy based solely on the inhibition of pro-angiogenic factors frequently has limitations, including the development of resistance and suboptimal therapeutic responses.

**Figure 4.**
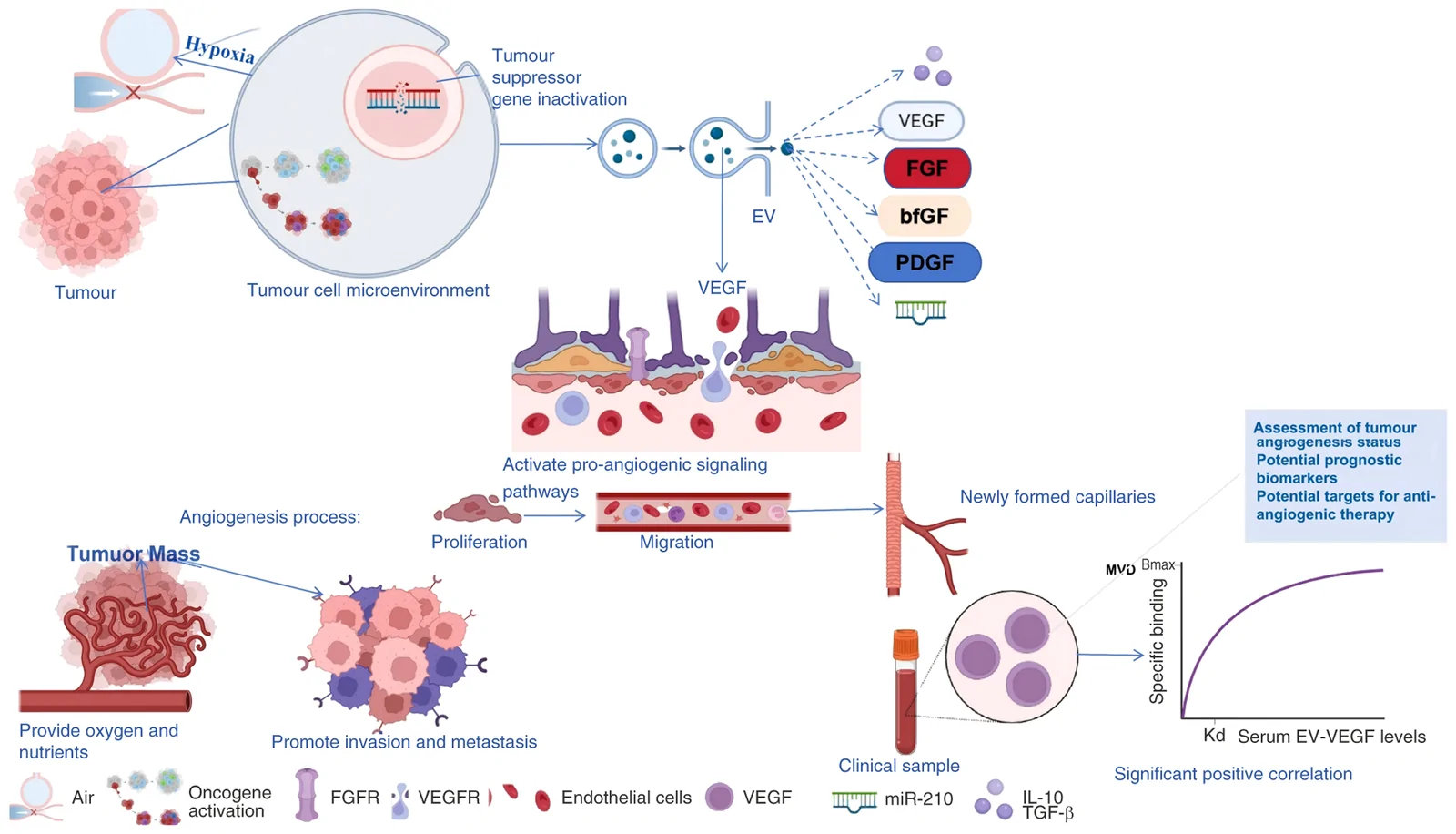
Schematic illustration of the mechanism by which EVs regulate tumour angiogenesis. Tumour-derived EVs are enriched in pro-angiogenic factors, such as VEGF and FGF, and deliver these factors to vascular endothelial cells. Subsequent activation of downstream signalling pathways promotes endothelial cell proliferation, migration and tube formation, providing material and nutritional support for tumour growth. EV, extracellular vesicle; VEGF, vascular endothelial growth factor; VEGFR, VEGF receptor; FGF, fibroblast growth factor; FGFR, FGF receptor; bFGF, basic fibroblast growth factor; PDGF, platelet-derived growth factor; TGF-β, transforming growth factor-β; MVD, microvessel density. Created using BioRender.

In conclusion, tumour-derived EVs trigger tumour angiogenesis through a multidimensional cascade involving cytokine activation, extracellular matrix remodelling and hypoxia-induced miRNA regulation, with multiple pro-angiogenic factors encapsulated within EVs exerting synergistic effects. However, the relative contributions of EVs and free pro-angiogenic factors to tumour angiogenesis remain unclear. Moreover, the angiogenic potency of EVs secreted by distinct cell populations requires further systematic investigation. Circulating EV-associated VEGF levels may serve as predictive biomarkers of the therapeutic efficacy of anti-angiogenic agents. Depletion of tumour-derived EVs or functional blockade of their pro-angiogenic cargo represents a novel therapeutic strategy for anti-angiogenic cancer treatment (Fig. 4).

### EV-mediated transport of key enzymes drives metabolic reprogramming and reshapes cellular energy metabolism

EVs act as central drivers of the horizontal transmission of metabolic reprogramming phenotypes and systematic remodelling of the tissue microenvironment. Metabolic reprogramming is widely recognised as a hallmark of cancer; it not only fuels malignant tumour cell proliferation through systematic rewiring of energy and biosynthetic metabolism, but also contributes to the establishment and maintenance of an immunosuppressive TME. The primary mechanism underlying tumour immune evasion involves the direct suppression of tumour-infiltrating immune cell function through competitive depletion of essential nutrients, including glucose and glutamine, and accumulation of immunosuppressive metabolic by-products, notably lactic acid, ultimately facilitating immune escape and tumour progression (52).

The core mechanism underlying EV-mediated metabolic reprogramming lies in the selective packaging and delivery of a broad panel of key metabolic enzymes regulated by transcription factors. Specifically, activation of key transcriptional regulators, such as HIF-1α and c-Myc, induces the expression of glucose transporter 1 and other membrane transport proteins, thereby markedly increasing cellular glucose uptake and supplying essential carbon substrates for downstream metabolic pathways (53). Subsequently, intracellular glucose is phosphorylated by hexokinase 2 (HK2), an enzyme consistently overexpressed in malignant tumours. This step commits glucose to glycolysis and serves as a critical determinant of glycolytic flux (54). Furthermore, tumour cells preferentially express the M2 isoform of pyruvate kinase (PKM2), which, through its distinct kinetic properties and non-metabolic regulatory functions, synergises with upstream alterations to establish a metabolic configuration conducive to rapid proliferation and biomass accumulation (55).

By simultaneously delivering key downstream metabolic enzymes, EVs function as central executors that drive the cascade amplification of glycolysis and the systemic acidification of the tissue microenvironment (56). Downstream of PKM2, lactate dehydrogenase A (LDHA) is upregulated as a pivotal metabolic enzyme that amplifies glycolytic flux, sustains high-rate glycolysis and drives substantial lactic acid accumulation, thereby providing an essential energetic substrate for rapid tumour cell proliferation (6). Lactic acid is actively exported from tumour cells through monocarboxylate transporters, not only eliminating metabolic waste but also inducing pronounced acidification of the TME. This acidification is not merely a passive by-product of heightened glycolytic activity. Rather, it functions as an active modulator that creates conditions favourable for tumour invasion, metastasis and immune evasion. Specifically, TME acidosis promotes tumour cell migration, local invasion and distant metastasis, while concurrently impairing anti-tumour immunity, most notably by suppressing cytotoxic T lymphocyte function through disruption of T-cell receptor signalling and critical metabolic pathways. Moreover, lactic acid recruits immunosuppressive cell populations, including Tregs and myeloid-derived suppressor cells, thereby reinforcing an immunosuppressive niche that facilitates tumour immune escape (57). This mechanistic framework has been experimentally validated in pancreatic ductal adenocarcinoma, in which pancreatic cancer-derived EVs deliver HK2 to recipient cells, resulting in ~2.5-fold increases in glucose uptake and lactate secretion (58). Collectively, this metabolic reprogramming establishes a localised, persistently acidic TME that critically supports tumour progression and immune evasion.

Regarding translational clinical applications, from a liquid biopsy perspective, HK2 and LDHA expression levels in EVs may serve as biomarkers for tumour metabolic subtyping and guide individualised therapy (59). For instance, patients with pancreatic cancer and high HK2 expression in circulating EVs show a better therapeutic response to glycolysis inhibitors such as 2-deoxyglucose. Meanwhile, serum EV-associated lactate levels may be used to assess TME acidification and predict immunotherapeutic efficacy. In therapeutic target selection, as described above, inhibition of HK2, delivered via pancreatic cancer cell-derived EVs, reduces glucose uptake in recipient cells by 62% and decreases lactate secretion by 71%, while markedly augmenting the activity of tumour-infiltrating T cells. Accordingly, targeting EV-mediated metabolic enzymes represents a viable strategy to abrogate metabolic reprogramming in the TME (60). With respect to engineered EVs, metabolic enzymes including HK2 and LDHA have become critical research targets. EVs loaded with glycolysis inhibitors enable precise targeting of tumour cell metabolic pathways, thereby avoiding systemic metabolic adverse effects. To date, engineered EVs encapsulating LDHA inhibitors have completed preclinical validation in pancreatic cancer models, demonstrating pronounced suppression of tumour growth and prolonged overall survival.

Collectively, these translational and pre-clinical observations highlight the central contribution of EV-packaged metabolic enzymes to tumour progression, prompting further mechanistic dissection of how tumour-derived EVs propagate metabolic reprogramming across the tumour microenvironment.

**Figure 5.**
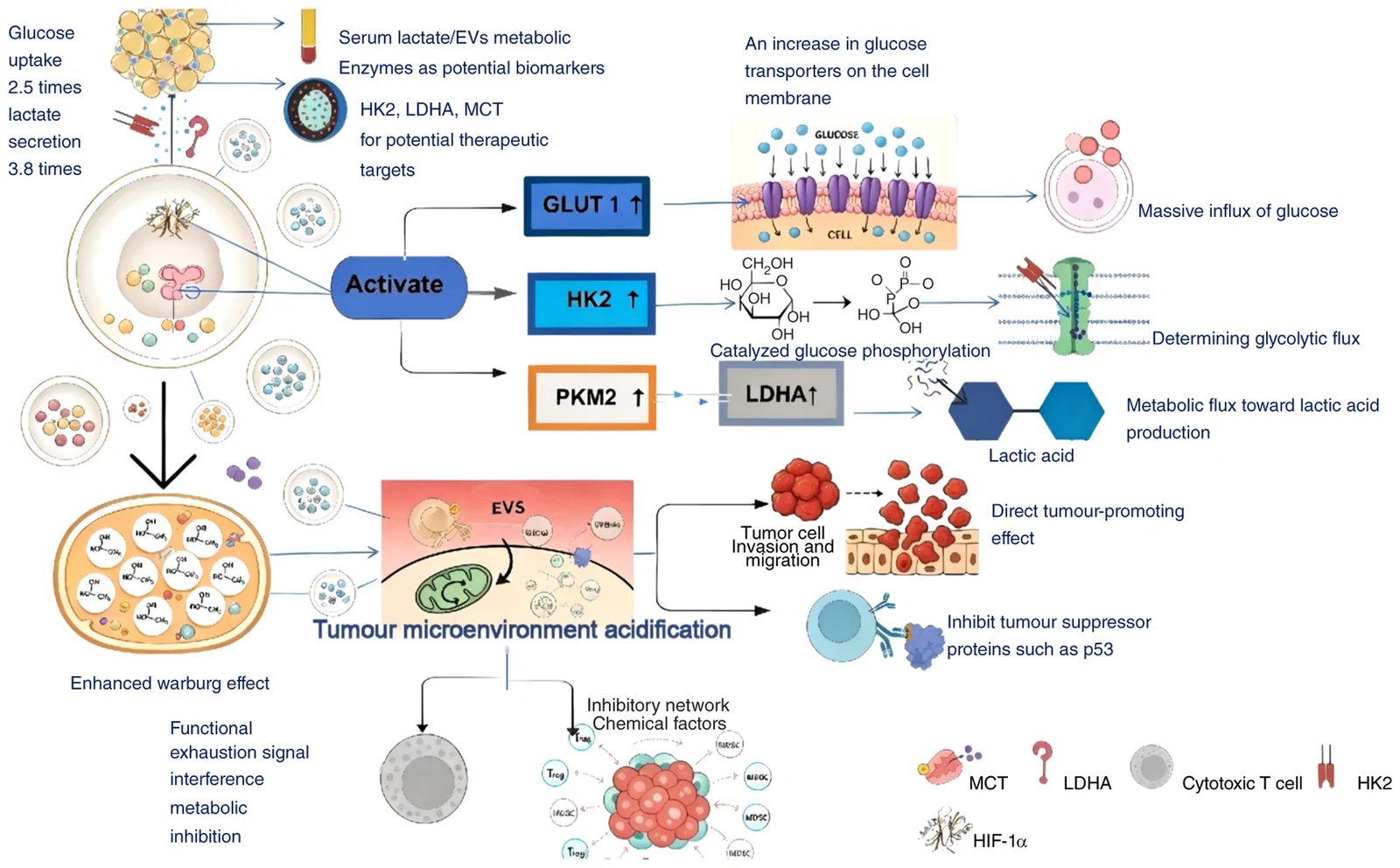
Schematic illustration of the mechanism of EV-mediated tumour metabolic reprogramming. Tumour-derived EVs deliver key glycolytic enzymes that trigger metabolic reprogramming in recipient cells. This process enhances glycolytic activity and induces tumour microenvironment acidification, thereby further accelerating tumour progression and immune evasion. The associated metabolic enzymes may serve as potential diagnostic and therapeutic targets. EV, extracellular vesicle; HK2, hexokinase 2; LDHA, lactate dehydrogenase A; MCT, monocarboxylate transporter; GLUT1, glucose transporter 1; PKM2, pyruvate kinase M2; p53, tumour protein p53; HIF-1α, hypoxia-inducible factor 1α. Created using BioRender.

The aforementioned studies demonstrate that key metabolic enzymes, particularly HK2 and LDHA, serve as core functional cargos of tumour-derived EVs and play pivotal roles in EV-mediated intercellular transmission of metabolic reprogramming by modulating cellular energy metabolism. Mediated by tumour-derived EVs, this metabolic reprogramming, notably characterised by enhanced glycolytic flux and increased lactate secretion, extends from individual tumour cells to the entire tissue niche, establishing a mechanistically coherent pathogenic axis linking intracellular metabolic alterations to systemic remodelling of the TME. This reprogramming exerts dual biological effects: First, it supplies the bioenergetic and biosynthetic substrates required for rapid tumour cell proliferation; second, it fosters an immunosuppressive TME through extracellular acidification, thereby facilitating immune evasion, a hallmark of malignant progression. Consequently, blocking the EV-mediated intercellular transfer of key metabolic enzymes, such as HK2 and LDHA, has emerged as a promising therapeutic strategy that can simultaneously suppress tumour metabolic reprogramming and the formation of an immunosuppressive microenvironment, exert dual anti-tumour effects, and offer novel mechanistic insights and potential avenues for clinical intervention in malignant tumours (Fig. 5).

### EVs mediate intercellular transfer of resistance-associated proteins, thereby fuelling therapeutic resistance

Multidrug resistance (MDR) in cancer markedly undermines the efficacy of clinical anticancer therapies (61). Accumulating evidence indicates that EVs, as critical vehicles for intercellular communication among tumour cells, contribute to MDR through multiple molecular mechanisms. Among these, multidrug resistance protein 1 (P-glycoprotein) has a central mechanistic role.

P-gp, a drug-resistance-associated transmembrane transporter, plays a pivotal role in mediating MDR in malignant tumours. Overexpression or hyperactivation of P-gp in tumour cells confers a survival advantage under chemotherapeutic pressure by facilitating the ATP-dependent efflux of diverse anticancer agents, thereby reducing intracellular drug accumulation and diminishing therapeutic efficacy (62). Furthermore, EVs secreted by drug-resistant tumour cells frequently exhibit elevated surface expression of clinically relevant biomarkers, including HER2 and CD20, that mirror those on the parental tumour cell membrane (63). In the context of targeted therapies such as trastuzumab (anti-HER2) and rituximab (anti-CD20), these EVs act as molecular decoys by competitively binding therapeutic antibodies, sequestering drug molecules and consequently lowering the bioavailable concentration of active drug reaching tumour cells, ultimately contributing to therapeutic resistance and clinical treatment failure (64).

The ABC transporter family represents a well-established molecular mechanism underlying MDR. Proteomic profiling has demonstrated notable upregulation of three key ABC transporters, ABCB1 (P-gp/MDR1), ABCC1 (MRP1) and ABCG2 (BCRP), in EVs isolated from drug-resistant tumours (65,66). These membrane-bound efflux pumps use energy derived from ATP hydrolysis to actively export chemotherapeutic agents from cells, thereby substantially diminishing intracellular drug accumulation and compromising therapeutic efficacy. Notably, MDR1 expression in EVs derived from paclitaxel-resistant ovarian cancer cell lines was ~5.8-fold higher than that observed in their drug-sensitive counterparts (67).

Moreover, EVs can deliver DNA damage repair-associated proteins, including topoisomerase IIα (TOP2A), to recipient cells. TOP2A is a critical regulator of DNA replication and cell proliferation, with enzymatic activity tightly coordinated with cell cycle progression. The expression of TOP2A peaks during the G_2_/M phase and declines rapidly after mitotic completion (68,69). Recent evidence has demonstrated that EV-mediated transfer of TOP2A disrupts canonical DNA damage repair mechanisms and confers a 3- to 4-fold increase in cisplatin resistance in HeLa cells (70).

Regarding clinical translational applications, for liquid biopsy biomarkers, as noted in previous studies, high preoperative serum EV expression of MDR1 in patients with ovarian cancer indicates a 5.8-fold elevated risk of paclitaxel resistance (71). EV-associated TOP2A levels can be used to predict the therapeutic response to cisplatin and guide the selection of postoperative adjuvant chemotherapy regimens (72). Targeting drug resistance-related proteins carried by EVs can reverse tumour therapeutic resistance. Combined administration of MDR1 inhibitors and EV-depletion agents increases intracellular paclitaxel concentration in drug-resistant ovarian cancer cells by 3.5-fold and restores chemosensitivity (73). Blocking EV-mediated intercellular transfer of TOP2A enhances the antitumour activity of cisplatin by 3- to 4-fold. For engineered EV applications, EVs loaded with drug resistance-reversing small interfering RNAs can specifically deliver gene-silencing molecules that target drug resistance (74). In multidrug-resistant breast cancer models, this strategy restores tumour cell sensitivity to doxorubicin to the level observed in drug-sensitive cell lines (75).

**Figure 6.**
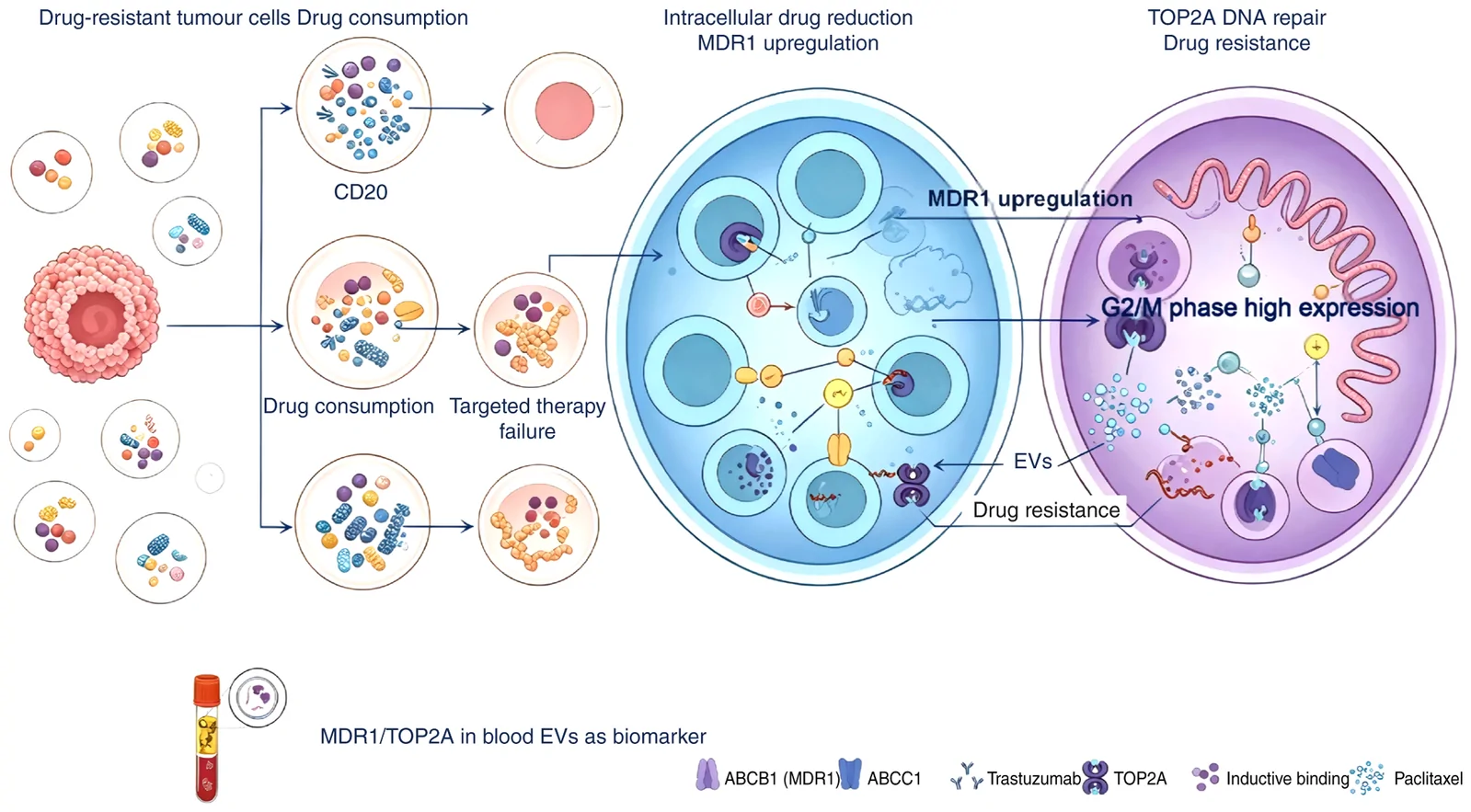
Schematic illustration of the mechanism of EV-mediated tumour multidrug resistance. EVs secreted Drug-resistant tumour cells secrete EVs that propagate drug resistance across tumour cell populations. Specifically, they deliver drug efflux transporters, DNA repair proteins and tumour surface molecules that mediate chemotherapeutic drug efflux, DNA repair and targeted drug neutralisation, respectively. Circulating EV-associated molecules can be used to predict the risk of drug resistance. EVs, extracellular vesicles; MDR1, multidrug resistance protein 1/ABCB1; TOP2A, DNA topoisomerase IIα; ABC, ATP-binding cassette. Created using BioRender.

In summary, from our perspective, drug resistance-associated proteins collectively contribute to tumour treatment resistance through multiple interconnected mechanisms, including pre-emptive drug binding, active efflux of chemotherapeutic agents and disruption of DNA repair pathways. These mechanisms establish a functional link between EV-mediated drug resistance and clinical treatment outcomes, positioning EVs as critical biomolecular carriers for investigating tumour drug resistance. Furthermore, they provide promising avenues for developing strategies to reverse resistance and optimise therapeutic regimens. Tumour-derived EVs confer therapeutic resistance via drug resistance proteins through drug binding, chemotherapeutic efflux and DNA repair disruption. However, the actual contribution of EV-mediated transmission of drug resistance to clinical tumour drug resistance remains unclear. Relevant studies remain limited, clinical data are insufficient and differences in transmission efficiency among distinct EV subpopulations during the propagation of drug resistance have not been fully elucidated. Detection of drug resistance-related protein expression levels in circulating EVs enables early prediction of tumour therapeutic response. The combined application of EV-depletion agents and conventional targeted agents is expected to overcome tumour drug resistance and improve therapeutic efficacy (Fig. 6; Table II).

## 3. Clinical applications of exosomes in malignant tumours

### Diagnostic and screening applications

Exosomes serve as promising biological biomarkers for liquid biopsy, exhibiting well-documented clinical utility in tumour screening and adjunctive diagnosis, progressing steadily from foundational research toward integration into clinical decision-making frameworks.

### Highly sensitive detection of protein biomarkers

Liquid biopsy platforms leveraging exosome-associated proteins demonstrate superior analytical sensitivity and specificity. Notably, immune checkpoint molecules, including PD-L1, which is consistently overexpressed on tumour-derived exosomes, represent robust and clinically relevant detection targets (76).

### Robust clinical diagnostic performance

Substantial clinical evidence substantiates the high diagnostic accuracy of exosome-based assays in prostate cancer detection. It is estimated that implementation of this technology could reduce unnecessary prostate biopsies by ~26%, thereby mitigating patient discomfort and minimizing wastage of healthcare resources associated with invasive procedures (77). This underscores the transformative potential of exosome-based liquid biopsy as a dynamic, non-invasive monitoring modality.

### Applications of therapeutic interventions

Exosomes function not merely as passive biomarkers but also as active therapeutic delivery vehicles and mechanistically relevant intervention targets. Their clinical translation is now advancing into the validation phase, particularly in addressing tumour drug resistance and improving therapeutic efficacy.

### Engineering targeted delivery systems

Exosomes engineered via surface conjugation of targeting ligands, such as EGFR-specific single-chain variable fragments, demonstrate markedly enhanced accumulation at tumour sites (78). This strategy offers a structurally grounded solution to two longstanding challenges in oncology: Suboptimal target specificity and dose-limiting systemic toxicity associated with conventional chemotherapeutics.

### Reprogramming the tumour immune microenvironment

Dual-targeting exosome-based inhibitors co-blocking PD-L1 and TGF-β have progressed to phase II clinical trial stage (79). Interim data indicate a ~38% reduction in the incidence of resistance to immune checkpoint blockade therapy (80). These findings substantiate that exosomes are not only integral components of resistance pathways but also promising, mechanism-informed therapeutic levers for overcoming immune evasion and reversing treatment resistance.

### Challenges in transformative manufacturing and quality assurance

Although exosomes hold considerable promise for clinical applications, their translation from laboratory research to large-scale clinical implementation is hindered by stringent industrial quality control requirements, constituting a critical bottleneck in their clinical translation.

## 4. Summary and prospects

The present review systematically elaborates on the core roles of EVs in the occurrence and development of malignant tumours, their clinical translational value, existing bottlenecks and future research directions.

EVs play a pivotal role in the core pathobiological mechanisms underlying malignant tumours. Specifically, EVs contribute to tumour progression through five well-documented functional axes: i) Metastasis-associated proteins that drive tumour cell invasion and dissemination; ii) immunosuppressive molecules that facilitate immune evasion; iii) pro-angiogenic factors that remodel the TME to support neovascularisation; iv) metabolic enzymes involved in cancer-specific metabolic reprogramming, thereby altering cellular energy metabolism; and v) drug resistance-associated proteins that confer resistance to conventional therapeutic agents.

A comprehensive investigation of the core mechanisms by which EVs influence malignant tumours has markedly advanced the understanding of tumour pathogenesis and established a robust theoretical foundation for developing EV-based liquid biopsy approaches and engineered therapeutic interventions.

Liquid biopsy technologies using EV-associated proteins have achieved substantial advances, particularly in the sensitive and specific detection of protein-based biomarkers, and show considerable clinical promise (64)

However, current research still faces notable challenges in addressing the functional heterogeneity of EV-associated proteins. First, EVs exhibit favourable biophysical and biological properties, including high biocompatibility, low immunogenicity and intrinsic structural stability, making them promising candidates for therapeutic applications (81). Second, these attributes confer distinct advantages over certain cell-based regenerative therapies, which are often associated with safety concerns, such as tumourigenicity and immune rejection, as well as practical limitations in scalability and standardisation. Although several EV-based therapeutics have advanced to clinical trials for disease management, translation to large-scale, good manufacturing practice-compliant production remains hindered by technical bottlenecks, including mechanical shear-induced damage during isolation and risks of microbial or co-isolated contaminant carryover (82). Furthermore, when EVs are deployed as biopharmaceutical agents or targeted delivery vehicles, rigorous quality control is indispensable, both to ensure precise dosing and batch-to-batch consistency, and to preserve EV integrity, stability and bioactivity after extensive purification. While engineering approaches, such as surface ligand conjugation and membrane modification, can enhance targeting specificity, comprehensive preclinical and clinical safety assessments remain essential. Critically, establishing standardised, physiologically relevant storage conditions that maintain both the structural fidelity and functional competence of EVs is essential to realise their full therapeutic potential (83).

Despite these challenges, research on the roles of EVs in malignant tumours remains a dynamic and promising field. We propose that future in-depth investigations of EVs in malignant tumours should focus on the following aspects: First, integrating multi-omics technologies with artificial intelligence approaches to construct heterogeneity atlases of EVs, thereby enabling precise identification and sorting of distinct functional subpopulations; second, establishing standardised systems for EV isolation, characterisation and quality control to accelerate the translation of basic research findings into clinical applications; and third, conducting systematic, well-designed clinical trials to comprehensively evaluate the clinical value of EVs in tumour diagnosis, prognostic assessment and therapy.

With continuous technological advances and a progressively deeper understanding of EV biology, EV research is expected to bring major breakthroughs in the precision diagnosis and treatment of malignant tumours, and in the advancement of liquid biopsy technologies.

## Figures and Tables

**Table I.** Classification of EV subpopulations and summary of cancer-specific diagnostic markers.

EV subpopulation	Biogenesis and basic characteristics	General reference markers	Cancer-specific markers (corresponding cancer types)	Cell type-specific markers (cellular origin)	Applicable cancer types	Clinical diagnosis and application scenarios
Exosomes (small EVs)	Endosomal origin; formed by inward budding of late endosomal membranes to generate MVBs, and released upon fusion of MVBs with the plasma membrane	Tetraspanins (CD9, CD63, CD81), ESCRT complex proteins (TSG101, Alix), heat shock proteins (HSP70, HSP90) and flotillin-1/2	GPC1 (pancreatic cancer), BARHL2 methylation (gastric cancer), miR-21/ miR-155 (pancreatic cancer), HER2 (breast cancer) and EGFR (lung cancer)	EpCAM (epithelial cell-derived), CD45 (immune cell-derived), ApoE/ApoB (hepatocyte- derived), CD41 (platelet- derived) and CD31 (endothelial cell-derived)	Pancreatic, gastric, breast, lung, ovarian and colorectal, prostate cancer	Non-invasive early cancer screening, minimal residual disease detection, recurrence monitoring, prognostic evaluation and adjuvant treatment regimen guidance
Microvesicles (large EVs/ectosomes)	Plasma membrane origin; formed by outward budding and fission of the plasma membrane; size, 100 nm-1 µm	Annexin A1, CD40L, PS, integrins and MMPs	MMP9 (melanoma), VEGFR2 (colorectal cancer), TF (pancreatic cancer), miR-122 (liver cancer) and CA125 (ovarian cancer)	CD44 (endothelial cell-derived), CD62P (platelet-derived), CD11b (myeloid cell-derived), CD14 (monocyte-derived) and cytokeratin 18 (CK18, epithelial cell-derived)	Melanoma, pancreatic, colorectal, lung, breast and ovarian cancer	Assessment of tumour invasive potential, prediction of cancer- associated thrombosis risk, metastasis risk assessment and evaluation of tumour microenvironment status
Apoptotic bodies	Apoptosis origin; released as membrane blebs from cells undergoing programmed cell death; size, 1–5 µm	Phosphatidylserine, MHC I/II, thrombospondin, LAMP1, histones and nuclear DNA fragments	Tumour-specific nuclear DNA mutations, Annexin V positivity/high phosphatidylserine expression, CK18 fragments (epithelial tumours) and M30 neoepitope (apoptotic levels tumour cells)	CK8/CK18 (epithelial cell-derived), CD45 (immune cell-derived), thrombospondin (endothelial cell-derived) and histones (nuclear origin)	Pan-cancer (especially solid tumours), haematological malignancies, lymphoma and leukaemia	Tumour burden monitoring, chemotherapy response evaluation, minimal residual disease detection and evaluation of therapy-induced apoptosis
Large oncosomes (tumour- specific large EVs)	Tumour cell plasma membrane origin; large, irregularly shaped vesicles shed by highly invasive tumour cells; size, 1–10 µm	Cytokeratins (CK18, CK19), CD44v6, integrins, myosin and actin	Oncogenic signalling proteins (AKT, Myc, Ras mutants), EGFRvIII (glioblastoma), androgen receptor splice variant 7 (prostate cancer) and miR-221 (aggressive breast cancer)	EpCAM/CK (tumour epithelial cell-derived), CD44 (cancer stem cell- derived) and vimentin (mesenchymal phenotype tumour cell-derived)	Prostate, breast and pancreatic cancer, glioblastoma and melanoma	Evaluation of tumour metastatic potential, assessment of circulating tumour cell status, detection of cancer stem cell activity and prediction of therapeutic efficacy

MVBs, multivesicular bodies; EVs, extracellular vesicles; MHC, major histocompatibility complex; EpCAM, epithelial cell adhesion molecule; miR, microRNA; ESCRT, endosomal sorting complexes required for transport; TSG101, tumour susceptibility gene 101; HSP, heat shock protein; MMP, matrix metalloproteinase; CK, cytokeratin; LAMP1, lysosomal-associated membrane protein 1.

**Table II.** Core mechanisms, key molecules and clinical translational applications of EVs in malignant tumours.

Core functional mechanisms	Key molecules/genes	Clinical applications in liquid biopsy	Clinical applications of engineered therapeutics	Validated clinical/experimental data
Mediating tumour invasion and organ-specific metastasis	Integrin αvβ3 (ITGAV/ITGB3), integrin α6β4 (ITGA6/ITGB4), integrin αvβ5 (ITGAV/ITGB5), MMP-14 (MMP14), ADAM10 (ADAM10), β1 integrin (ITGB1)	Risk stratification of tumour metastasis; prediction of organotropic metastasis tendency; dynamic monitoring of postoperative recurrence	Targeted blockade of integrins on the EV surface to inhibit pre-metastatic niche formation; engineered EVs loaded with matrix remodelling inhibitors to block local tumour invasion	Integrin αvβ3 carried by breast cancer-derived EVs is markedly associated with lung metastasis risk; MMP-14 expression in cervical cancer-derived EVs is positively associated with tumour stage and depth of invasion
Mediating tumour immune escape	PD-L1 (CD274) and TGF-β (TGFB1)	Prediction of immunotherapeutic efficacy; assessment of immune escape risk; prognostic stratification of patients	Development of PD-L1/TGF-β dual-targeted EV inhibitors to block immunosuppressive signalling; engineered EVs loaded with immune activators to reverse the local immunosuppressive microenvironment	EV inhibitors targeting the PD-L1/ TGF-β dual axis have entered phase II clinical trials, reducing the incidence of immunotherapy resistance by ~38%; serum EV PD-L1 levels in patients with melanoma are negatively associated with the response rate to anti-PD-1 therapy
Driving tumour angiogenesis	VEGF (VEGFA), bFGF (FGF2), PDGF (PDGFA/B), IL-8 (CXCL8), miR-210 (MIR210)	Assessment of tumour angiogenic activity	Targeted elimination of pro- angiogenic EVs to disrupt tumour nutrient supply; engineered EVs loaded with anti-angiogenic drugs to precisely suppress neovascularization	Serum EV–VEGF levels in patients with colorectal cancer are markedly positively associated with tumour tissue MVD scores; EV–VEGF concentration in cerebrospinal fluid of patients with glioblastoma is markedly elevated relative to normal levels
Remodelling tumour energy metabolism and microenvironmental acidification	HK2 (HK2), LDHA (LDHA), GLUT1 (SLC2A1), PKM2 (PKM2), MCT1 (SLC16A1)	Assessment of tumour metabolic activity; grading of tumour malignancy; prediction of patient overall survival	Blockade of EV-mediated metabolic enzyme transfer to suppress tumour glycolytic flux; engineered EVs loaded with metabolic inhibitors to reverse the acidic immunosuppressive microenvironment	HK2 delivered by pancreatic cancer cell-derived EVs increases glucose uptake capacity of recipient cells by ~2.5-fold and lactate secretion by ~3.8-fold; serum EV-LDHA levels are markedly negatively associated with overall survival in patients with pancreatic cancer
Mediating tumour therapeutic resistance	MDR1 (ABCB1), ABCC1 (ABCC1), ABCG2 (ABCG2), TOP2A (TOP2A), HER2 (ERBB2) and CD20 (MS4A1)	Early prediction of chemotherapy/ targeted therapy resistance; basis for dynamic adjustment of treatment regimens; real-time monitoring of therapeutic efficacy	Targeted elimination of drug resistance-associated EVs to reverse tumour multidrug resistance; engineered EVs loaded with chemotherapeutic/ genetic drugs to overcome tumour drug resistance barriers; blockade of competitive binding between EVs and targeted agents	MDR1 expression in EVs from paclitaxel-resistant ovarian cancer cell lines is ~5.8-fold that in drug- sensitive strains; EV-mediated TOP2A transfer increases cisplatin resistance of tumour cells by 3–4 folds; detection of EV protein biomarkers in prostate cancer helps avoid ~26% of unnecessary biopsies
Mediating tumour invasion and organ-specific metastasis	Integrin αvβ3 (ITGAV/ITGB3), integrin α6β4 (ITGA6/ITGB4), integrin αvβ5 (ITGAV/ITGB5), MMP-14 (MMP14), ADAM10 (ADAM10) and β1 integrin (ITGB1)	Risk stratification of tumour metastasis; prediction of organotropic metastasis tendency; dynamic monitoring of postoperative recurrence	Targeted blockade of integrins on the EV surface to inhibit pre-metastatic niche formation; engineered EVs loaded with matrix remodelling inhibitors to block local tumour invasion	Integrin αvβ3 carried by breast cancer-derived EVs is markedly associated with lung metastasis risk MMP-14 expression in cervical cancer-derived EVs is positively associated with tumour stage and depth of invasion

EVs, extracellular vesicles; MVD, micro vessel density; LDHA, lactate dehydrogenase; HK2, hexokinase 2; PD-L1, programmed death-ligand 1; ADAM10, a disintegrin and metalloproteinase 10; MMP, matrix metalloproteinase; bFGF, basic fibroblast growth factor; PDGF, platelet-derived growth factor, miR-, microRNA; GLUT1, glucose transporter 1; PKM2, M2 isoform of pyruvate kinase; MCT1, monocarboxylate transporter 1; MDR1, multidrug resistance 1; TOP2S, topoisomerase IIα.

## Data Availability

Not applicable.
